# Allogeneic hematopoietic cell transplantation with cord blood versus mismatched unrelated donor with post-transplant cyclophosphamide in acute myeloid leukemia

**DOI:** 10.1186/s13045-021-01086-2

**Published:** 2021-05-03

**Authors:** Bhagirathbhai Dholaria, Myriam Labopin, Jaime Sanz, Annalisa Ruggeri, Jan Cornelissen, Hélène Labussière-Wallet, Didier Blaise, Edouard Forcade, Patrice Chevallier, Anna Grassi, Ludmila Zubarovskaya, Jürgen Kuball, Patrice Ceballos, Fabio Ciceri, Frederic Baron, Bipin N. Savani, Arnon Nagler, Mohamad Mohty

**Affiliations:** 1 Department of Hematology-Oncology, Vanderbilt University Medical Center, 220 Pierce Ave, 777 Preston Research Building, Nashville, TN 37232 USA; 2 EBMT ALWP Office, Hôpital Saint-Antoine, Paris, France; 3 Hematology Department, University Hospital La Fe, Valencia, Spain; 4 Department of Pediatric Hematology and Oncology IRCCS, Ospedale Pediatrico Bambino Gesù, Rome, Italy; 5 Department of Hematology, Erasmus MC Cancer Institute, University Medical Center Rotterdam, Rotterdam, The Netherlands; 6 Hôpital Lyon Sud, Hospices Civils de Lyon, Pierre Bénite, France; 7 Programme de Transplantation and Therapie Cellulaire, Centre de Recherche en Cancérologie de Marseille, Institut Paoli Calmettes, Marseille, France; 8 Hôpital Haut-Leveque, CHU Bordeaux, Pessac, France; 9 Department of D’Hematologie, CHU Nantes, Nantes, France; 10 Hematology and Bone Marrow Transplant Unit, ASST Papa Giovanni XXIII, Bergamo, Italy; 11 RM Gorbacheva Research Institute, Pavlov University, St. Petersburg, Russian Federation; 12 Department of Haematology, University Medical Centre, Utrecht, The Netherlands; 13 Département d’Hématologie Clinique, CHU Lapeyronie, Montpellier, France; 14 Ospedale San Raffaele S.R.L., Haematology and BMT, Milan, Italy; 15 CHU and University of Liège, Liège, Belgium; 16 Chaim Sheba Medical Center, Tel Hashomer, Israel; 17 ALWP Office Hôpital Saint-Antoine, Paris, France; 18 Service d’Hématologie Clinique et Thérapie Cellulaire, Hôpital Saint-Antoine, UMRs 938, AP-HP, Sorbonne University, and INSERM, Paris, France

**Keywords:** Mismatched donor, Cord blood transplantation, Cord blood unit, Acute leukemia, Acute myeloid leukemia, Toxicity, Graft-versus-host disease, Disease relapse, Allogeneic hematopoietic cell transplantation, Peripheral blood stem cell, Bone marrow, Post-transplant cyclophosphamide, Human leukocyte antigen

## Abstract

**Background:**

Allogeneic hematopoietic cell transplantation (allo-HCT) using a mismatched unrelated donor (MMUD) and cord blood transplantation (CBT) are valid alternatives for patients without a fully human leukocyte antigen (HLA)-matched donor. Here, we compared the allo-HCT outcomes of CBT versus single-allele-mismatched MMUD allo-HCT with post-transplant cyclophosphamide (PTCy) in acute myeloid leukemia.

**Methods:**

Patients who underwent a first CBT without PTCy (*N* = 902) or allo-HCT from a (HLA 9/10) MMUD with PTCy (*N* = 280) were included in the study. A multivariate regression analysis was performed for the whole population. A matched-pair analysis was carried out by propensity score-based 1:1 matching of patients (177 pairs) with known cytogenetic risk.

**Results:**

The incidence of grade II–IV and grade III–IV acute graft-versus-host disease (GVHD) at 6 months was 36% versus 32% (*p* = 0.07) and 15% versus 11% (*p* = 0.16) for CBT and MMUD cohorts, respectively. CBT was associated with a higher incidence of graft failure (11% vs. 4%, *p* < 0.01) and higher 2-year non-relapse mortality (NRM) (30% vs. 16%, *p* < 0.01) compared to MMUD. In the multivariate analysis, CBT was associated with a higher risk of, NRM (HR = 2.09, 95% CI 1.46–2.99, *p* < 0.0001), and relapse (HR = 1.35, 95% CI 1–1.83, *p* = 0.05), which resulted in worse leukemia-free survival (LFS) (HR = 1.68, 95% CI 1.34–2.12,* p* < 0.0001), overall survival (OS) (HR = 1.7, 95% CI 1.33–2.17, *p* < 0.0001), and GVHD-free, relapse-free survival (GRFS) (HR = 1.49, 95% CI 1.21–1.83, *p* < 0.0001) compared to MMUD. The risk of grade II–IV acute GVHD (*p* = 0.052) and chronic GVHD (*p* = 0.69) did not differ significantly between the cohorts. These results were confirmed in a matched-pair analysis.

**Conclusions:**

CBT was associated with lower LFS, OS, and GRFS due to higher NRM, compared to MMUD allo-HCT with PTCy. In the absence of a fully matched donor, 9/10 MMUD with PTCy may be preferred over CBT.

**Supplementary Information:**

The online version contains supplementary material available at 10.1186/s13045-021-01086-2.

## Introduction

Allogeneic hematopoietic cell transplantation (allo-HCT) is commonly offered to patients with acute myeloid leukemia (AML) as a curative treatment modality. The degree of human leukocyte antigen (HLA) matching between recipient and donor has long been considered an important factor impacting allo-HCT outcomes. A higher degree of HLA allele and/or antigen mismatch leads to a higher risk of graft-versus-host disease (GVHD) and non-relapse mortality (NRM); hence, an 8/8 HLA-matched related or unrelated donor is preferred over an HLA-mismatched donor. Most patients from ethnic minority groups do not have a fully HLA-matched (HLA-A, B, C, DRB1 loci match) unrelated donor [1]. The donor options for patients without a fully HLA-matched donor are haploidentical-related (haplo) donor, mismatched unrelated donor (MMUD), or umbilical cord blood transplantation (CBT).

CBT using HLA-mismatched single or double cord blood units has shown good leukemia-free survival (LFS) and overall survival (OS) in patients with AML [2–5]. In registry-based studies, the incidence and severity of acute and chronic GVHD have been lower with mismatched CBT compared to what has previously been reported in recipients of fully or partially HLA-matched related or unrelated donor marrow transplantation [6–9]. Across these studies, CBT was associated with relatively higher NRM driven by delayed engraftment and immune reconstitution, resulting in comparable LFS and OS compared to 8/8 or 7/8 HLA-matched unrelated donor transplantation [10]. Although increasing HLA disparity among patients receiving CBT is associated with a higher risk of GVHD [11], the incidence is still lower than with unrelated donor with a similar degree of HLA disparity. Many advances have been made in peri-transplant immunomodulation to overcome the HLA barrier between the recipient and donor. The use of post-transplant cyclophosphamide (PTCy) pioneered by the group at Johns Hopkins was originally developed in patients receiving allo-HCT from a haplo donor and bone marrow (BM) graft [12, 13]. PTCy reduces the risk of GVHD by inducing alloreactive T cell dysfunction and promoting graft tolerance [14]. Over the past decade, PTCy has been rapidly incorporated across the donor types and stem cell graft sources with GVHD and relapse incidence rates comparable to historical calcineurin inhibitor (CNI)-based GVHD prophylaxis regimens [15–21]. Reduced GVHD-related NRM has been reported with MMUD in the setting of PTCy compared to historical antithymocyte globulin (ATG)-based transplantation [18, 22, 23]. A prospective phase II study by the National Marrow Donor Program/Be The Match (NMDP/BTM) showed that outcomes using 4–7/8 MMUD in the setting of PTCy were similar to those obtained using a haplo donor [24].

A comparison of the two available alternative donor sources, CBT and single-allele MMUD, in the setting of PTCy has not been described so far. In this study, we compared the allo-HCT outcomes of CBT versus 9/10 MMUD with PTCy in patients with AML.

## Methods

### Study design and data collection

This was a retrospective multicenter analysis using the dataset of the Acute Leukemia Working Party (ALWP) of the European Society for Blood and Marrow Transplantation (EBMT) registry. The EBMT is a voluntary working group of more than 600 transplant centers that are required to report all consecutive stem cell transplantations and follow-ups once a year. Audits are routinely performed to determine the accuracy of the data. The eligibility criteria for this analysis included adult patients ≥ 18 years of age with AML who underwent a first allo-HCT using CBT or MMUD with PTCy between 2010 and 2019. The MMUD was defined as an unrelated donor with single HLA-allele mismatch at one of the following HLA loci A, B, C, DRB1, or DQB1. CBT using single or double cord blood units without PTCy were included in the analysis. The exclusion criteria were allo-HCT from any other donor source; a previous history of allo-HCT; use of ex vivo graft manipulation; or lack of information on HLA matching or GVHD prophylaxis. We also excluded six patients with a pre-transplant disease status of third complete remission found only in the MMUD group. Data collected included recipient and donor characteristics [age, gender, and cytomegalovirus (CMV) serostatus], baseline Karnofsky performance status (KPS), disease features and status at transplant, year of transplant, type of conditioning regimen, stem cell source, GVHD prophylaxis regimen, and the use of in vivo T cell depletion (TCD). The conditioning regimen was defined based on the reports from individual transplant centers as per previously established EBMT criteria [25]. In addition to PTCy in the MMUD cohort, other immunosuppressive drugs were used as per institutional protocols. All patients received 50 mg/kg × 2 doses (either Day + 3 and + 4 or day + 3 and + 5). There was heterogeneity in schedule other immunosuppressive drugs based on institutional practice [26]. Grading of acute GVHD was performed using established criteria [27]. Chronic GVHD was classified as limited or extensive according to published criteria [28]. A modified GVHD-free, relapse-free survival (GRFS) was defined as previously described [29]. For this study, all necessary data were collected according to the EBMT guidelines, using the EBMT minimum essential data forms. The list of institutions reporting data included in this study is provided in Additional file 1: Table S1.

### Ethics approval and consent to participate

The study was approved by the scientific board of the ALWP of the EBMT. The study protocol was approved by each site and complied with country-specific regulatory requirements. All patients gave informed consent to use their personal information for research purposes. The study was conducted as per the Declaration of Helsinki and Good Clinical Practice guidelines.

### Statistical analysis

The study endpoints were OS, LFS, relapse incidence (RI), NRM, engraftment, acute and chronic GVHD incidence, and GRFS. All endpoints were measured from the time of transplantation. OS was defined as time to death from any cause. LFS was defined as survival with no evidence of relapse or progression. We used modified GRFS criteria, and GRFS events were defined as the first event among grade III–IV acute GVHD, extensive chronic GVHD, relapse, and death from any cause [29, 30].

Patient-, disease-, and transplant-related characteristics were compared between the two donor groups (CBT vs. MMUD) using the Mann–Whitney test for numerical variables, and the Chi-square or Fisher’s exact test for categorical variables. The probabilities of OS, LFS, and GRFS were calculated using the Kaplan–Meier (KM) estimate. The RI and NRM were calculated using cumulative incidence curves in a competing risk setting, death in remission being treated as a competing event for relapse. The median follow-up duration was calculated using the reverse KM method where the event is being alive, and death is censored. Death was considered as a competing event for engraftment. To estimate the CI of acute or chronic GVHD, relapse and death were considered as competing events. Univariate analyses were carried out using the log-rank test for LFS and OS, while Gray’s test was used for CI. Multivariate analyses were performed with the Cox proportional hazards regression models. In the final Cox model, variables differing significantly between the two groups or potential risk factors were included. We did not adjust for the variable related to selection of donor, donor sex, donor CMV status, and TCD. To test for a center effect, we introduced a random effect or ‘frailty’ for each center into the model [31]. A matched-pair analysis was conducted using data only from patients with information on cytogenetics to better understand the association between donor source and allo-HCT outcomes. The propensity score was based on recipient age, recipient gender, cytogenetics, disease status before transplant, conditioning intensity, and KPS at transplantation. Exact matching for cytogenetics, disease status and recipient gender and nearest neighbor for KPS and conditioning were used. Caliper width was 0.20. Patients well matched with standardized mean difference estimates of less than 5% for all parameters were included in the propensity score. Exact matching for cytogenetics, disease status, and recipient gender, and nearest neighbor for KPS and conditioning were used. Caliper width was 0.20 of the standard deviation of the logit of the estimated propensity score. All *p* values were two-sided with a type 1 error rate fixed at 0.05. Statistical analyses were performed with SPSS 24.0 (SPSS Inc, Chicago, IL, USA) and R 4.0.3 [R Core Team (2020). R: A language and environment for statistical computing. R Foundation for Statistical Computing, Vienna, Austria. URL https://www.R-project.org/].

### Data sharing statement

Please contact the EBMT for the raw data used for this study (www.ebmt.org).

## Results

### Patient, transplant, and disease characteristics

Baseline patient, transplant, and disease characteristics between the study cohorts are shown in Table 1. A total of 902 patients in the CBT and 280 patients in the MMUD cohort met the study inclusion criteria. The median follow-up duration from allo-HCT was longer for the recipients of CBT compared to MMUD (46.8 vs. 19.1 months, *p* < 0.01). As the adoption of PTCy was relatively recent, the median year of transplant for the MMUD cohort was 2017, whereas it was 2013 for CBT recipients. The proportion of allele mismatches was 38% for HLA-A, 20% for HLA-B, 19% for HLA-C, 8% for HLA-DRB1, and 15% for HLA-DQB1 locus in MMUD cohort. Baseline KPS, hematopoietic cell transplantation comorbidity index (HCT-CI), and recipient age were not statistically different between the study cohorts.

**Table 1:** Baseline patient, disease, and transplant characteristics

	MMUD (*N* = 280)	CBT (*N* = 902)	*p* value
*Follow-up (reverse KM, months)*			
Median (IQR)	19.1 (11.4–36.7)	46.8 (22.6–72.6)	< 0.001
*Patient age (years)*			
Median (min–max) [IQR]	52.1 (18.2–75.6) [39.9–61.2]	50.5 (18.1–73.2) [38.4–60.3]	0.087
*Year transplant*			
Median (min–max) [IQR]	2017 (2010–2019)	2013 (2010–2019)	< 0.001
*Cytogenetics risk group*^*a*^			
Good risk	18 (6.4%)	50 (5.5%)	0.001
Intermediate risk	148 (52.9%)	366 (40.6%)	
Adverse risk	40 (14.3%)	144 (16%)	
Unknown risk	74 (26.4%)	342 (37.9%)	
*Disease status at transplantation*			
CR1	179 (63.9%)	522 (57.9%)	0.023
CR2 +	49 (17.5%)	230 (25.5%)	
Advanced	52 (18.6%)	150 (16.6%)	
*Patient gender*			
Male	163 (58.2%)	430 (47.7%)	0.002
*Donor gender*			
Male	187 (68.2%)	421 (50%)	< 0.001
Missing	6	60	
*Female donor to male recipient*			
	35 (12.7%)	194 (22.2%)	< 0.001
Missing	5	29	
*HCT-CI*			
0	125 (59%)	294 (57.3%)	0.073
1 or 2	32 (15.1%)	112 (21.8%)	
≥ 3	55 (25.9%)	107 (20.9%)	
Missing	68	389	
*KPS score*			
≥ 90	76 (27.1%)	210 (23.3%)	0.19
*Patient CMV serostatus: positive*			
Positive	201 (73.6%)	587 (66.9%)	0.036
Missing	7	24	
*Donor CMV serostatus: positive*			
Positive	127 (45.8%)	260 (35.2%)	0.002
Missing	3	163	
*Graft source*			
	BM—19 (6.8%)	Single unit—408 (45.2%)	
	PBSC—261 (93.2%)	Double unit—494 (54.8%)	
*Conditioning regimen intensity*			
MAC	141 (50.4%)	416 (46.1%)	0.21
RIC	139 (49.6%)	486 (53.9%)	
*Type of conditioning regimen*			
BuCy	14 (5%)	23 (2.5%)	< 0.001
BuFlu	116 (41.4%)	14 (1.6%)	
TBF	45 (16.1%)	263 (29.2%)	
TBI-based	38 (13.6%)	522 (57.9%)	
Other	67 (23.9%)	80 (8.9%)	
*GVHD prophylaxis*^*b*^			
csa + mmf	111 (39.6%)	638 (70.7%)	
tacro + mmf	51 (18.2%)	22 (2.4%)	
csa + mtx	10 (3.6%)	35 (3.9%)	
csa	34 (12.1%)	183 (20.3%)	
tacro	24 (8.6%)	2 (0.2%)	
siro + mmf	14 (5%)	8 (0.9%)	
other	36 (12.9%)	14 (1.6%)	

*CMV* cytomegalovirus; *GVHD* graft-versus-host disease, *KPS* Karnofsky performance status, *HCT-CI* hematopoietic cell transplantation comorbidity index, *KM* Kaplan–Meier, *IQR* interquartile rage, *BM* bone marrow, *PBSC* peripheral blood stem cell, *MAC* myeloablative conditioning, *RIC* reduced-intensity conditioning, *BuCy* busulfan cyclophosphamide, *BuFu* busulfan, fludarabine, *TBF* thiotepa, busulfan, fludarabine, *TBI* total body irradiation, *CSA* cyclosporine, *mtx* methotrexate, *mmf* mycophenolate mofetil, *tacro* tacrolimus, *siro* sirolimus

^a^Per UK MRC criteria

^b^All MMUD patients received PTCy

The majority (93%) of MMUD transplants were performed using mobilized peripheral blood stem cell (PBSC) grafts and 55% of CBT were performed with combined two cord units. Information about graft composition was available in a subset of patients. In CBT cohort, the median total nucleated cell dose was 0.37 × 10^6^ cells/kg [interquartile range (IQR) 0.24–0.50] and CD34 cell dose was 0.11 × 10^6^ cells/kg (IQR 0.06–0.19). In MMUD cohort, the median total nucleated cell dose was 7.7 × 10^6^ cells/kg (IQR 5.32–10.01) and CD34 cell dose was 6.0 × 10^6^ cells/kg (IQR 4.5–7.9). More patients in the CBT cohort received in vivo TCD using ATG or alemtuzumab, compared to MMUD (in addition to PTCy) (40% vs. 26%, *p* < 0.01). More details on in vivo TCD are provided in Additional file 1: Table S2. Cyclosporin with mycophenolate mofetil (MMF) was the common GVHD prophylaxis regimen in both cohorts (71% in CBT and 40% in MMUD). Myeloablative conditioning (MAC) was used in nearly half of transplants (46% in CBT and 50% in MMUD, *p* = 0.21). Busulfan plus fludarabine was used in 41% of MMUD patients, and TBI-based conditioning was used in 58% of CBT patients. Information on the graft composition (total nucleated cell, CD34 and CD3 cell counts) between the study cohort**s** was not available for the majority of the study patients.

### Engraftment

The median time to ANC engraftment was 23 days for CBT versus 19 days for MMUD (*p* < 0.001). The cumulative incidence of an absolute neutrophil count (ANC) above 500 cells/µL at 30 days after transplantation was 67% in CBT versus 92% in MMUD (*p* = 0.001). Cumulative incidence of platelets above 20,000 cells/µL at day 90 was 77% in CBT versus 91% in MMUD (*p* = 0.0001). Graft failure or loss (including death before engraftment) was reported for 95 (10.4%) patients who received CBT and 12 (4.4%) patients who received MMUD allo-HCT (*p* = 0.002).

### GVHD

The incidence of grade II–IV acute GVHD during the first 180 days was 36% in CBT patients compared to 31.6% in MMUD patients (*p* = 0.07). The cumulative incidence of grade III–IV acute GVHD did not differ (CBT-14.7% vs. MMUD-11.4%, *p* = 0.16) between the cohorts. The 2-year incidence of overall (CBT-26.2% vs. MMUD-31.5%, *p* = 0.20) and extensive (CBT-11.6% vs. MMUD-11.6%, *p* = 0.83) chronic GVHD was similar between the study cohorts. In the multivariate analysis (Table 2), there was no statistically significant difference in the risk of grade II–IV acute GVHD [hazard ratio (HR) = 1.32, 95% confidence interval (CI): 1.0–1.74, *p* = 0.052] and chronic GVHD (HR = 0.94, 95% CI 0.68–1.30, *p* = 0.69) between CBT versus MMUD. We also ran another multivariate analysis using three groups: 9/10 MMUD, single CBT, and double CBT (Additional file 1: Table S5). Double CBT was associated higher risk of grade II–IV acute GVHD compared to MMUD. Acute GVHD risk was comparable between single CBT versus MMUD.

**Table 2:** Multivariate analysis of outcomes based on patient, disease, and transplant characteristics

Variable	Grade II–IV, acute GVHD		Chronic GVHD		Relapse		NRM		LFS		OS		GRFS	
	HR (95% CI)	*p* value	HR (95% CI)	*p* value	HR (95% CI)	*p* value	HR (95% CI)	*p* value	HR (95% CI)	*p* value	HR (95% CI)	*p* value	HR (95% CI)	*p* value
*Donor source*														
MMUD	1.00		1.00		1.00		1.00		1.00		1.00		1.00	
CBT	1.32 (1–1.74)	0.052	0.94 (0.68–1.3)	0.69	1.35 (1–1.83)	0.05	2.09 (1.46–2.99)	< 0.0001	1.68 (1.34–2.12)	< 0.0001	1.7 (1.33–2.17)	< 0.0001	1.49 (1.21–1.83)	0.0002
Patient age (per 10 years)	1.11 (1.02–1.21)	0.02	1.06 (0.96–1.17)	0.24	0.99 (0.9–1.08)	0.81	1.31 (1.18–1.45)	< 0.001	1.12 (1.05–1.2)	< 0.001	1.19 (1.11–1.28)	< 0.001	1.07 (1.01–1.14)	0.02
*Pre-HCT disease status*														
CR1	1.00		1.00		1.00		1.00		1.00		1.00		1.00	
CR2	1.05 (0.81–1.35)	0.72	1.08 (0.8–1.45)	0.63	1.07 (0.78–1.48)	0.67	1.3 (0.97–1.74)	0.08	1.2 (0.97–1.48)	0.10	1.26 (1.01–1.58)	0.04	1.14 (0.94–1.38)	0.20
Advanced	0.78 (0.57–1.08)	0.13	0.95 (0.61–1.5)	0.84	2.89 (2.19–3.83)	< 0.001	2.03 (1.47–2.8)	< 0.001	2.42 (1.96–2.99)	< 0.001	2.57 (2.07–3.21)	< 0.001	1.81 (1.48–2.21)	< 0.001
*Cytogenetics*														
Good risk	1.00		1.00		1.00		1.00		1.00		1.00		1.00	
Intermediate risk	1.22 (0.75–2)	0.42	0.75 (0.45–1.24)	0.26	1.59 (0.77–3.31)	0.21	0.81 (0.48–1.35)	0.41	1.09 (0.72–1.65)	0.69	1.07 (0.69–1.66)	0.75	0.94 (0.66–1.34)	0.73
Adverse risk	1.37 (0.8–2.34)	0.25	0.69 (0.37–1.26)	0.22	3.78 (1.79–7.98)	< 0.001	0.75 (0.41–1.36)	0.34	1.74 (1.12–2.71)	0.01	1.48 (0.93–2.36)	0.10	1.36 (0.92–1.99)	0.12
Unknown risk	1.32 (0.79–2.19)	0.29	0.88 (0.52–1.5)	0.64	1.92 (0.92–4)	0.08	0.85 (0.5–1.44)	0.54	1.23 (0.81–1.88)	0.33	1.2 (0.77–1.87)	0.42	1.18 (0.82–1.7)	0.36
*Donor gender*														
Male donor	1.00		1.00		1.00		1.00		1.00		1.00		1.00	
Female donor	0.82 (0.67–1.01)	0.06	0.9 (0.7–1.15)	0.40	0.94 (0.74–1.2)	0.62	0.87 (0.69–1.11)	0.26	0.9 (0.76–1.06)	0.22	0.91 (0.76–1.08)	0.29	0.83 (0.71–0.97)	0.02
*Conditioning regimen*														
MAC	1.00		1.00		1.00		1.00		1.00		1.00		1.00	
RIC	0.89 (0.69–1.14)	0.34	0.86 (0.64–1.16)	0.33	1.27 (0.97–1.64)	0.08	0.6 (0.46–0.8)	< 0.001	0.86 (0.71–1.03)	0.10	0.79 (0.65–0.97)	0.02	0.85 (0.71–1.01)	0.06
KPS ≥ 90	0.74 (0.58–0.94)	0.02	1.19 (0.86–1.64)	0.30	0.95 (0.72–1.24)	0.69	0.8 (0.6–1.05)	0.10	0.87 (0.72–1.05)	0.16	0.86 (0.7–1.05)	0.15	0.85 (0.71–1.02)	0.09
Centre (frailty)		< 0.001		< 0.001		0.24		0.03		0.30		0.18		0.10

*CBT* cord blood transplant, *MMUD* mismatched unrelated donor, *CR* complete remission, *GVHD* graft-versus-host disease, *NRM* non-relapse mortality, *PFS* progression-free survival, *OS* overall survival, *GRFS* GVHD-relapse-free survival, *KPS* Karnofsky performance scale, *RIC* reduced intensity conditioning, *MAC* myeloablative conditioning, *HR* hazard ratio, *CI* confidence interval

### Relapse, NRM, and survival

There was no statistically significant difference in 2-year RI (CBT-27.5% vs. MMUD-23.2%, *p* = 0.24), but 2-year NRM was significantly higher in CBT compared to MMUD (CBT-29.7% vs. MMUD-16.3%, *p* = 0.001). In the univariate analysis, 2-year LFS (CBT-42.8% vs. MMUD-60.5%, *p* = 0.001), OS (CBT-46.8% vs. MMUD-62.8%, *p* = 0.001), and GRFS (CBT-33.9% vs. MMUD-46.8%, *p* = 0.001) were significantly lower in the recipients of CBT compared to MMUD allo-HCT, respectively (Additional file 1: Table S3).In the multivariate analysis (Table 2), there was no significant statistical difference in RI (HR = 1.35, 95% CI 1.0–1.83, *p* = 0.05) between CBT versus MMUD. CBT was associated with higher risk of NRM (HR = 2.09, 95% CI 1.46–2.99, *p* < 0.0001) compared to MMUD after adjusting for other patient- and transplant-related factors. The CBT was associated with poorer LFS (HR = 1.68, 95% CI 1.34–2.12 *p* < 0.0001), OS (HR = 1.70, 95% CI 1.33–2.17, *p* < 0.0001), and GRFS (HR = 1.49, 95% CI 1.21–1.83 *p* = 0.0002) compared to MMUD. The use of a reduced-intensity conditioning (RIC) regimen was associated with higher RI (HR = 1.27, 95% CI 0.97–1.64, *p* = 0.08), lower NRM (HR = 0.60, 95% CI 0.46–0.80, *p* < 0.001), and better OS (HR = 0.79, 95% CI 0.65–0.97, *p* = 0.02) compared to a MAC regimen. Other factors associated with poor OS were older age and CR2 or advanced disease status before transplantation as shown in Table 2. MMUD was associated with better survival when the Cox model was adjusted for TCD and year of transplant (data not shown). In the separate Cox analysis (Additional file 1: Table S5), relapse risk was lower with double CBT compared to single CBT but not significantly different from MMUD. Other HCT outcomes were better with 9/10 MMUD with less NRM and improved LFS, OS, and GRFS compared to single/double CBT.

### Matched-pair analysis

**Fig. 1:**
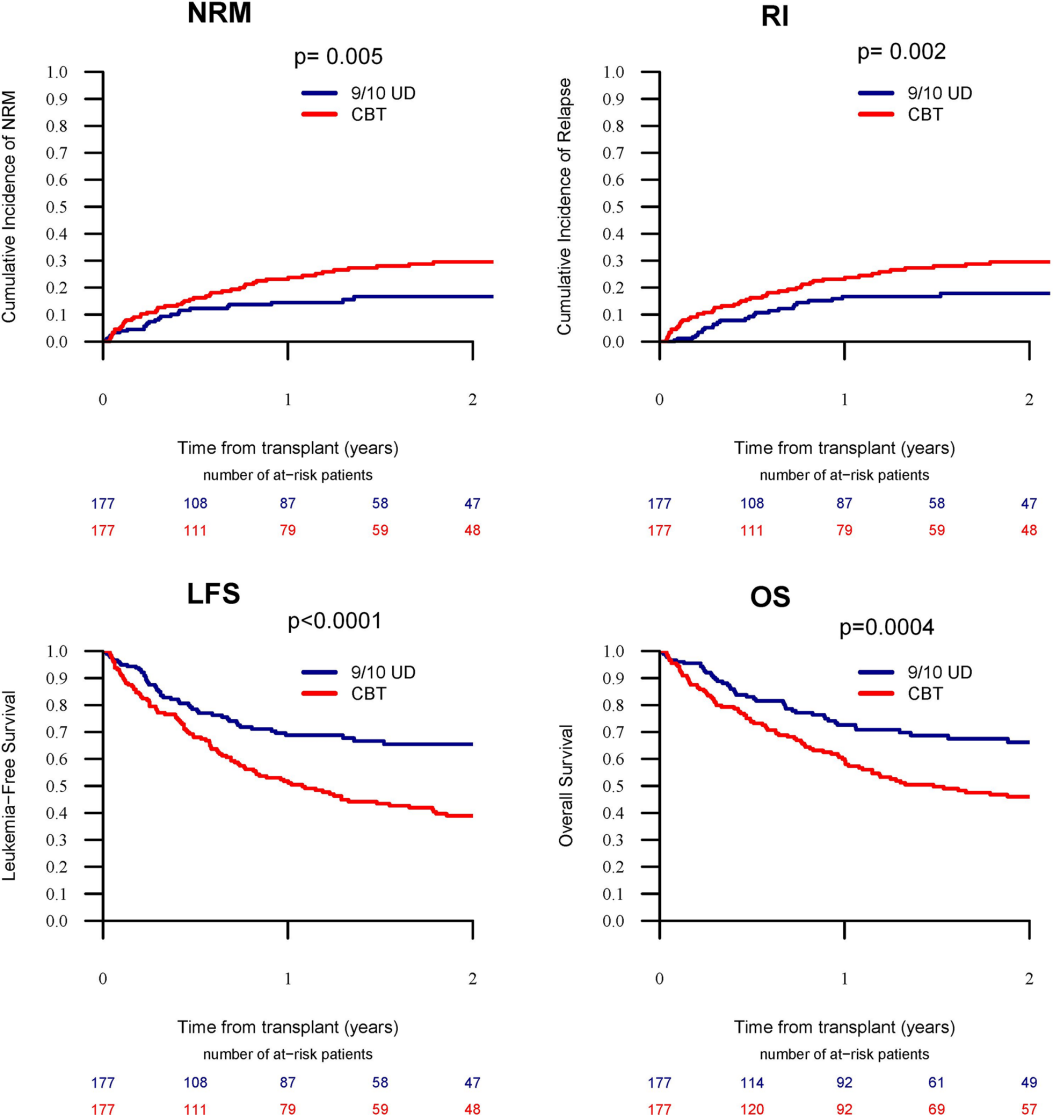
Transplant outcomes between CBT versus 9/10 MMUD with PTCy (matched pairs). *NRM* non-relapse mortality, *RI *relapse incidence, *LFS* leukemia-free survival, *OS* overall survival

**Fig. 2:**
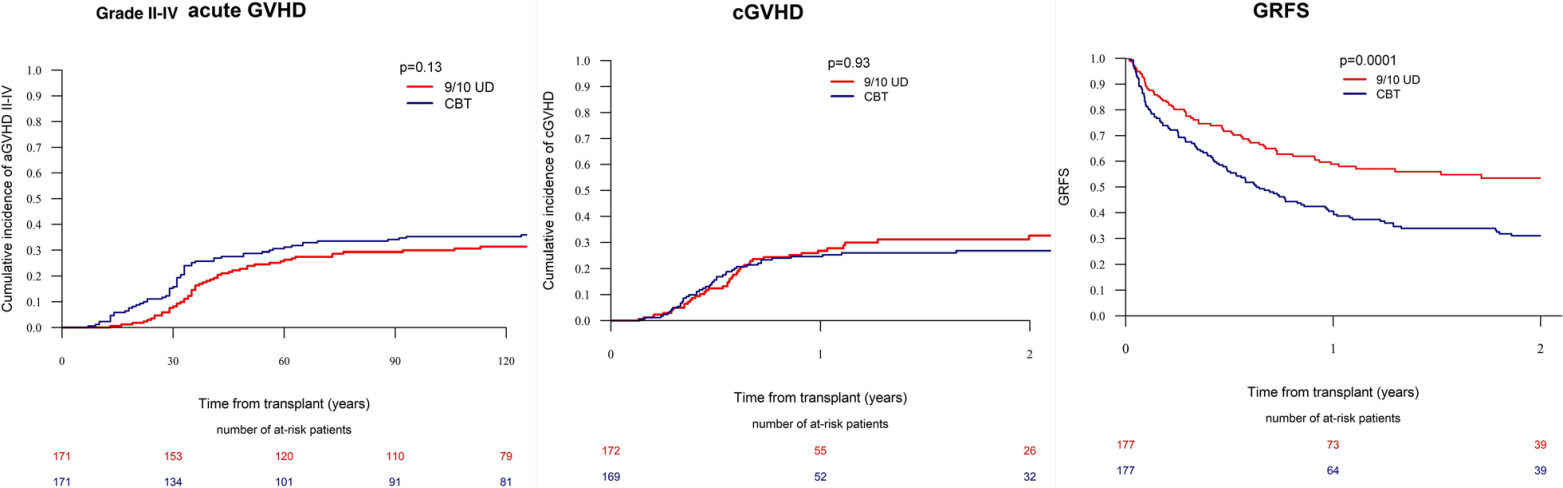
Graft-versus-host disease (GVHD) and GVHD-free, relapse-free survival (GRFS)

Next, we performed a matched-pair analysis of patients with complete cytogenetic information (Additional file 1: Table S4). A total of 177 pairs (CBT: MMUD = 1:1) were selected according to the criteria described in the methods section. CBT was associated with higher 2-year RI (31.5% vs. 17.9%, *p* = 0.002) and NRM (29.5% vs. 16.7%, *p* = 0.005), resulting in lower LFS (38.9% vs. 65.4%, *p* < 0.001), OS (46% vs. 66.2%, *p* < 0.001), and GRFS (31% vs. 53.5%, *p* < 0.001) compared to MMUD (Figs. 1, 2). The incidence of acute and chronic GVHD was comparable between the two cohorts.

### Toxicity

A total of 441 (49%) patients in the CBT and 84 (30%) patients in the MMUD cohort died during the study period (Table 3). Disease relapse was the most common cause of death in both cohorts (36% of deaths). More patients in the CBT cohort died from infection compared to MMUD (30.9% vs. 24.1%). GVHD-related deaths were comparable (16.2% vs. 14.5%). There were five (1.2%) deaths due to lymphoproliferative disease and three (0.7%) deaths due to graft failure in the CBT cohort and none in the MMUD cohort due to these complications.

**Table 3:** Major cause of death

Etiology	MMUD (*N* = 84)	Cord blood transplant (*N* = 441)
Original disease	30 (36.1%)	155 (36%)
Infection	20 (24.1%)	133 (30.9%)
Graft-versus-host disease	12 (14.5%)	70 (16.2%)
Cardiac toxicity	1 (1.2%)	1 (0.2%)
Hemorrhage	1 (1.2%)	10 (2.3%)
Graft failure/rejection	0 (0%)	3 (0.7%)
Veno-occlusive disease	3 (3.6%)	9 (2.1%)
Interstitial pneumonitis	6 (7.2%)	13 (3%)
Lymphoproliferative disorder	0 (0%)	5 (1.2%)
Second malignancy	1 (1.2%)	8 (1.9%)
Multiorgan failure	6 (7.2%)	11 (2.6%)
Other transplant related	3 (3.6%)	11 (2.6%)
Missing cause of death	1	12

## Discussion

Alternative donor transplantation is a life-saving procedure for those patients with AML who lack a fully HLA-matched donor. Our study showed that allo-HCT using a single-allele (HLA-9/10) MMMD with PTCy resulted in better LFS, OS, and GRFS compared to CBT. The improvement in survival from the use of MMUD was likely due to lower NRM compared to CBT in our study cohorts. CBT was associated with a higher incidence of graft failure- and infection-related deaths.

PTCy has shown the capacity to overcome the HLA barrier by rapidly stimulating regulatory T cells (Tregs), specifically alloreactive Tregs [14]. Many centers have adopted the original Hopkins haplo-BM PTCy protocol for MMUD allo-HCT. The retrospective studies of MMUD allo-HCT in the setting of PTCy showed a lower risk of GVHD and NRM compared to historical ATG or alemtuzumab-based MMUD allo-HCT [18, 22, 32–34]. Compared to ATG or alemtuzumab, PTCy is associated with better immune reconstitution and hence lower risk of infection-related NRM [35]. A recent EBMT study by Battipaglia et al. showed a lower risk of acute GVHD and better LFS and GRFS with PTCy compared to ATG in patients who underwent allo-HCT from 9/10 MMUD [18]. The NMDP conducted a prospective phase II study of MMUD BM transplantation with PTCy. One-year OS for the entire cohort was 76%. The survival outcomes were comparable to the contemporaneous cohort of haplo-HCT from the Center for International Blood and Marrow Transplant Research [24]. These results show that outcomes of MMUD allo-HCT can be improved significantly by incorporation of PTCy.

Partially HLA-matched CBT is used preferentially at many centers in the absence of a fully matched donor. There are certain advantages of CBT, such as rapid availability, increased tolerance to HLA mismatches, and lower risk of GVHD compared to HLA-mismatched marrow or PBSC graft. However, CBT is associated with higher NRM secondary to delayed engraftment and immune reconstitution compared to other donor sources. Although outcomes from the centers with CBT expertise are excellent, the number of patients undergoing CBT is declining due to preferential use of haplo donors with PTCy [36]. The Blood and Marrow Transplant Clinical Trials Network (BMT CTN 1101) prospectively compared double cord transplant versus haplo/PTCy after a RIC regimen. Although LFS was comparable between the groups, higher NRM associated with CBT resulted in lower OS compared to haplo/PTCy [37]. Previous registry-based studies have shown comparable survival after CBT versus 7/8 MMUD with conventional GVHD prophylaxis [3]. Our analysis showed superior NRM with MMUD compared to CBT. These results are likely due to faster engraftment, lower infection-related death, and graft failure with MMUD compared to CBT. Since MMUD patients were transplanted relatively recently compared to CBT, improved supportive care over time may have a positive impact on NRM. PTCy allows safe early discontinuation of immunosuppression after the graft infusion [38], which may have resulted in better immune reconstitution compared to CBT. This information was not available in our database. The interaction of ATG use on the outcomes of CBT versus MMUD PTCy cohort is more complex. ATG has shown to impair immune reconstitution, resulting in higher risk of viral infections and NRM after CBT [39, 40]. In pediatric setting, the use of low dose ATG was associated with better T cell reconstitution and event-free survival [41]. A poor reconstitution of natural killer (NK) cells and conventional T cells after haplo-HCT with PTCy compared to HLA-matched allo-HCT has been reported [42], but a similar effect in the setting of MMUD with PTCy needs to be proven. Compared to PTCy alone, combination of low-dose ATG and PTCy appears to delay T cell reconstitution but support a quicker reconstitution of some NK cells subtypes after haplo PBSC transplantation [43]. This combination appears to reduce the risk of GVHD without increasing NRM in the setting of haplo and unrelated donor PBSC transplantation [44–46]. We had a subset of patients (26.4%) who received dual in vivo TCD with PTCy and ATG in MMUD cohort, but main study outcomes were unchanged when the Cox model was adjusted for in vivo TCD (data not shown).

Multiple strategies are under investigation to improve the safety and efficacy of CBT. E*x vivo* expansion of cord blood units [47–49] and co-infusion of haploidentical PBSC grafts with cord blood units [50] may allow faster engraftment and reduce the risk of graft failure with CBT. The single-center retrospective analysis by Milano et al. showed that CBT may offer a better graft-versus-leukemia (GVL) effect and lower the risk of relapse in patients with measurable residual disease (MRD) before transplantation compared to unrelated donor transplantation [51]. Unfortunately, information about MRD was not available for most patients included in the study. The results showed a higher RI with CBT in the matched-pair analysis which may suggest an ability of PTCy to induce graft tolerance and preserve the GVL effect [14].

This analysis was limited by the retrospective nature of the study. Our inability to adjust for unknown or unmeasured factors may have affected the transplantation outcomes. Heterogeneity in conditioning regimens, GVHD prophylaxis, center volume, and supportive therapy may have affected the study outcomes. The information on National Institute of Health chronic GVHD grading, MRD, donor chimerism, graft composition, and recipient antibody status against HLA allele mismatch was missing in a subset of the included patients. This is the first comparative analysis of these two alternative donor sources and confirms that outcomes of MMUD transplantation may be improved with the incorporation of PTCy.

## Conclusion

In this registry-based study, we showed that CBT was associated with a higher NRM and lower survival compared to allo-HCT from 9/10 MMUD with PTCy in patients with AML. In the absence of a fully HLA-matched or haplo donor, MMUD with PTCy may be preferred over CBT. A prospective study with uniform conditioning and TCD is required to validate our results.

## Supplementary Information

**Additional file 1.** Supplementary material.

## Data Availability

Please contact the EBMT for the raw data used for this study (www.ebmt.org).
