# Supplementary material for: Allogeneic hematopoietic cell transplantation with cord blood versus mismatched unrelated donor with post-transplant cyclophosphamide in acute myeloid leukemia
Source: J Hematol Oncol. 2021 May 3;14:76. doi: 10.1186/s13045-021-01086-2 (PMC8094558; doi:10.1186/s13045-021-01086-2)
Supplement: Supplementary file 1 — Additional file 1. Supplementary material. [file 13045_2021_1086_MOESM1_ESM.docx]

Supplementary material

Table s1: center list

| **CENTRES** | **N** |
| --- | --- |
| 663 Valencia [H Univ La Fe] | 68 |
| 246 Rotterdam [Erasmus MC] | 57 |
| 671 Lyon [H E Herriot] | 48 |
| 230 Marseille [Paoli Calmettes] | 32 |
| 267 Pessac [H Haut-Leveque] | 32 |
| 253 Nantes [Hotel Dieu] | 29 |
| 658 Bergamo [Ospedale, ematol] | 27 |
| 813 Milano [S Raffaele] | 26 |
| 725 St._Petersburg [Pavlov Med Univ] | 25 |
| 239 Utrecht [University] | 24 |
| 926 Montpellier [University] | 24 |
| 218 London [Royal Marsden] | 23 |
| 240 Bologna [S Orsola-Malpighi] | 23 |
| 717 Nottingham [City H] | 22 |
| 214 Barcelona [H Clinic] | 21 |
| 656 Prague [Ist Hematology] | 21 |
| 386 Bristol [Royal H Sick Chil] | 20 |
| 260 Barcelona [SCreu i S Pau] | 19 |
| 270 Grenoble [H A Michallon] | 17 |
| 304 Firenze [Careggi-Meyer] | 17 |
| 203 Leiden [Univ H] | 14 |
| 308 Graz [Medical Univ] | 13 |
| 387 Birmingham [Queen Elizabeth] | 13 |
| 588 Amsterdam [VU Univ Med Ctr] | 13 |
| 665 Clamart [H Percy] | 13 |
| 775 Paris [St Antoine] | 13 |
| 206 Copenhagen [Rigshospitalet] | 12 |
| 227 Vienna [Medizinische Univ] | 12 |
| 264 Poitiers [H La Miletrie] | 12 |
| 277 Lille [H Claude Huriez] | 12 |
| 251 Caen [Hopital, Hematol] | 11 |
| 233 Besancon [H Jean Minjoz] | 10 |
| 305 Torino [Regina Margherita] | 10 |
| 624 Toulouse [H Purpan] | 10 |
| 756 Rome [Tor Vergata] | 10 |
| 207 Paris [St Louis] | 9 |
| 217 Genova [S Martino] | 9 |
| 523 Nice [H de l`ARCHET I] | 9 |
| 601 Manchester [Royal Infirmary] | 9 |
| 666 Villejuif [Gustave Roussy] | 9 |
| 941 Rouen [Becquerel] | 9 |
| 202 Basel [202] | 8 |
| 252 Creteil [H Mondor Hematol] | 8 |
| 254 Leeds [St James] | 8 |
| 546 Groningen [Univ H] | 8 |
| 613 Barcelona [H Trias i Pujol] | 8 |
| 614 Hamburg [Univ H] | 8 |
| 763 London [Kings College H] | 8 |
| 558 Munich [Rechts der Isar] | 7 |
| 584 Barcelona [V d`Hebron Adults] | 7 |
| 676 Vandoeuvre_Les_Nancy [Hosp] | 7 |
| 780 Manchester [Christie] | 7 |
| 787 Regensburg [University] | 7 |
| 235 Oslo [Rikshospitalet] | 6 |
| 606 Cuneo [S Croce e Carle] | 6 |
| 650 Angers [CHRU] | 6 |
| 672 Strasbourg [H Hautepierre] | 6 |
| 754 Tel-Hashomer [Univ Adults] | 6 |
| 809 Erlangen [University] | 6 |
| 930 Moscow [NRC Haem.] | 6 |
| 153 Hamburg [AK St Georg] | 5 |
| 212 Stockholm [Univ H] | 5 |
| 250 Saint_Etienne [St Etienne] | 5 |
| 258 Jerusalem [Univ Hadassah] | 5 |
| 273 Clermont-Ferrand [Jean Perrin] | 5 |
| 287 Rome [S C-Forlanini] | 5 |
| 354 Milano [Trap Mid Osseo] | 5 |
| 513 Munich [Kl Grosshadern] | 5 |
| 565 Maastricht [Univ H] | 5 |
| 617 Ankara [Ibni Sina H] | 5 |
| 710 Perth [Royal H] | 5 |
| 727 Salamanca [H Clinico] | 5 |
| 810 Freiburg [University] | 5 |
| 977 Limoges [CHRU] | 5 |
| 237 Nijmegen [St Radboud] | 4 |
| 244 Glasgow [Royal Infirmary] | 4 |
| 282 Valencia [H Clinico] | 4 |
| 409 Petach-Tikva [Beilinson H] | 4 |
| 556 Budapest [National Med Ctr] | 4 |
| 659 Brest [C.H.R.U Brest] | 4 |
| 661 Rennes [H Sud/Pontchaillou] | 4 |
| 778 Sheffield [Royal Hallamshire] | 4 |
| 919 Antalya [Medic Park H] | 4 |
| 208 Zürich [208] | 3 |
| 209 Leuven [Univ H] | 3 |
| 224 London [UCL] | 3 |
| 234 Brussels [St. Luc] | 3 |
| 255 Oxford [Radcliffe H] | 3 |
| 294 Milano [Osp Niguarda] | 3 |
| 361 La_Coruña [Juan Canalejo] | 3 |
| 392 Palermo [Osp V Cervello] | 3 |
| 713 Leicester [Royal Infirmary] | 3 |
| 722 Palma_De_Mallorca [Son Dureta] | 3 |
| 726 Liege [University] | 3 |
| 731 Umeå [Univ H] | 3 |
| 744 Gent [Univ H] | 3 |
| 819 Madrid [H G Marañón] | 3 |
| 141 Brescia [Civili, Adulti] | 2 |
| 161 Tel_Aviv [Sourasky] | 2 |
| 211 Sao_Paulo [H Sirio-Libanes] | 2 |
| 231 Torino [S. Giovanni (CTO)] | 2 |
| 238 Córdoba [Reina Sofia] | 2 |
| 261 Geneva [261] | 2 |
| 266 Uppsala [Univ H] | 2 |
| 281 Patras [Univ H] | 2 |
| 283 Lund [Univ H] | 2 |
| 284 Birmingham [Heartlands H] | 2 |
| 295 Hannover [Medical Univ] | 2 |
| 309 Madrid [Jiménez Díaz] | 2 |
| 345 Haifa [Rambam MCH] | 2 |
| 397 Riyadh [King Faisal] | 2 |
| 428 Gliwice [Sklodowska] | 2 |
| 440 Kocaeli [Anadolu] | 2 |
| 559 Granada [V de las Nieves] | 2 |
| 566 Cambridge [Addenbrookes H] | 2 |
| 630 Brussels [Univ H] | 2 |
| 642 Oviedo [H Covadonga] | 2 |
| 645 Marburg [Philipps Univ] | 2 |
| 692 Palermo [La Maddalena] | 2 |
| 735 Murcia [H M Meseguer] | 2 |
| 769 Sevilla [Virgen del Rocio] | 2 |
| 788 Ancona [Umberto I] | 2 |
| 996 Antwerp_Edegem [UZA] | 2 |
| 160 Paris [H Necker] | 1 |
| 215 Brussels [Jules Bordet] | 1 |
| 225 Turku [University] | 1 |
| 242 Santander [Valdecilla] | 1 |
| 257 Dublin [St James] | 1 |
| 262 Paris [Pitie-Salpetriere] | 1 |
| 265 Milano [Osp Maggiore] | 1 |
| 276 Newcastle-Upon-Tyne [Freeman H] | 1 |
| 286 Pavia [S Matteo] | 1 |
| 289 Goeteborg [Sahlgrenska Univ H] | 1 |
| 299 Bolzano [Osp S Maurizio] | 1 |
| 301 Marseille [Timone, Oncologie] | 1 |
| 307 Rome [Univ S Cuore] | 1 |
| 321 Siena [Le Scotte] | 1 |
| 332 Taranto [Osp Nord] | 1 |
| 338 Halle [Univ Martin-Luther] | 1 |
| 352 Utrecht [Maxima Ped Onc] | 1 |
| 367 Luebeck [Schleswig-Holstein] | 1 |
| 421 Vigo [Comp H Univ] | 1 |
| 424 Montréal [H S Justine] | 1 |
| 434 Singapore [Gen H] | 1 |
| 444 Riyadh [Aziz] | 1 |
| 526 San_Giovanni_Rotondo [IRCCS] | 1 |
| 533 Jena [Friedrich-Schiller] | 1 |
| 539 London [St George`s] | 1 |
| 544 Monza [Osp S Gerardo] | 1 |
| 561 Thessaloniki [Papanicolaou G H] | 1 |
| 576 Málaga [H Carlos Haya] | 1 |
| 612 Cádiz [H del SAS] | 1 |
| 615 Madrid [H Ramón y Cajal] | 1 |
| 623 Verona [Policlinico] | 1 |
| 704 Southampton [General H] | 1 |
| 711 Sydney [Child H Westmead] | 1 |
| 712 Wuerzburg [Medizinische Kl II] | 1 |
| 734 Madrid [La Paz] | 1 |
| 740 Linköping [Univ H] | 1 |
| 768 London [S Bartholomew`s] | 1 |
| 785 Homburg [Univ Saarland] | 1 |
| 825 Alessandria [SS Ant e Bia] | 1 |
| 931 Suzhou [First Soochow] | 1 |
| 963 Lille [J de Flandre] | 1 |
| 994 Istanbul [Nightingale] | 1 |
| ***Total*** | ***1182*** |

Table s2: Details on *in vivo* T cell depletion used in each study cohort

|  | **MMUD, N (%)** | **CBT, N (%)** |
| --- | --- | --- |
| no *in vivo* TCD | 206 (73.6%) | 543 (60.2%) |
| *in vivo* TCD | 74 (26.4%) | 359 (39.8%) |
|  |  |  |
| ATG | 66 | 359 |
| Alemtuzumab | 8 | 0 |
| **ATG dose distribution** | | |
| if Thymoglobulin^$^ | n=37 | n=237 |
| 2,5 to 4 mg/kg | 2 | 22 |
| 4 to 6 mg/Kg | 22 | 72 |
| 6 to 7,5 mg/Kg | 3 | 113 |
| >7,5 mg/Kg | 10 | 30 |
| missing dose | 37 |  |
|  |  |  |
| if Fresenius^$^ | n=11 | n=29 |
| 16 to 25 mg/Kg | 2 | 9 |
| 25 to 35 mg/Kg | 0 | 9 |
| 35 to 45 mg/Kg | 5 | 2 |
| >45 mg/Kg | 4 | 9 |
|  |  |  |
| missing brand & dose | 18 | 93 |

^$^ Exact information about brand of ATG was missing in the registry. Above table assumes that any dose <16 mg/kg were Thymoglobulin and ≥16 mg/kg were ATG-Fresenius. The results should be interpreted with caution.

Abbreviations: TCD- T cell depletion, ATG- antithymocyte globulin.

Table s3: Univariate analysis of transplant outcomes

| **Outcomes** | 180 days | | 2 years | | | | | | |
| --- | --- | --- | --- | --- | --- | --- | --- | --- | --- |
|  | **Acute GVHD II-IV** | **Acute GVHD III-IV** | **chronic GVHD** | **ext. chronic GVHD** | **Relapse** | **NRM** | **LFS** | **OS** | **GRFS** |
| MMUD | 31.6%[26.1-37.2] | 11.4%[8-15.6] | 31.5%[25.1-38] | 11.6%[7.5-16.6] | 23.2%[17.9-29] | 16.3%[11.9-21.3] | 60.5%[53.7-66.6] | 62.8%[55.8-69] | 46.8%[40-53.3] |
| CBT | 36%[32.8-39.2] | 14.7%[12.4-17.1] | 26.2%[23.1-29.3] | 11.6%[9.5-14] | 27.5%[24.5-30.6] | 29.7%[26.6-32.8] | 42.8%[39.4-46.2] | 46.8%[43.3-50.2] | 33.9%[30.6-37.1] |
| p-value | 0.07 | 0.16 | 0.20 | 0.83 | 0.24 | 0.001 | 0.001 | 0.001 | 0.001 |

Abbreviations: NRM-nonrelapse mortality; LFS-leukemia-free survival; OS-overall survival; GVHD- graft-vs-host disease; GRFS-GVHD, relapse free survival

Table s4: Match pair analysis of transplant outcomes between CBT and MMUD allo-HCT

| **Outcomes** | **180 days** | | **2 years** | | | | | | |
| --- | --- | --- | --- | --- | --- | --- | --- | --- | --- |
|  | **Acute GVHD grade II-IV** | **Acute GVHD grade III-IV** | **chronic GVHD** | **Extensive chronic GVHD** | **Relapse** | **NRM** | **LFS** | **OS** | **GRFS** |
| MMUD | 31.3%[24.4-38.4] | 10.5%[6.5-15.7] | 32.6%[24.3-41] | 12.6%[7.3-19.5] | 17.9%[12-24.7] | 16.7%[11-23.3] | 65.4%[56.8-72.8] | 66.2%[57.2-73.7] | 53.5%[44.6-61.6] |
| CBT | 35.9%[28.7-43.1] | 14.7%[9.9-20.5] | 26.8%[20.1-34] | 13.4%[8.6-19.3] | 31.5%[24.4-38.9] | 29.5%[22.6-36.7] | 38.9%[31.2-46.6] | 46%[38-53.6] | 31%[24-38.3] |
| HR (95% CI) (cluster = pairs)  CBT (reference- MMUD) | 1.33 (0.92-1.93) | 1.5 (0.82-2.76) | 1.02 (0.65-1.61) | 1.46 (0.73-2.91) | 2.01 (1.28-3.14) | 1.96 (1.23-3.12) | 1.98 (1.44-2.72) | 1.82 (1.3-2.54) | 1.77 (1.32-2.37) |
| p value | 0.13 | 0.19 | 0.93 | 0.29 | 0.002 | 0.005 | < 0.0001 | 0.0004 | 0.0001 |

Abbreviations: CTB- cord blood transplantation; MMUD- mismatched unrelated donor; NRM-nonrelapse mortality; LFS-leukemia-free survival; OS-overall survival; GVHD- graft-vs-host disease; GRFS-GVHD, relapse free survival; HR-hazard ratio; CI-confidence interval

Table S5: Cox regression analysis including single versus double CBT

|  | **RELAPSE** | | **NRM** | **LFS** | | | **OS** | | **Acute GVHD II-IV** | | **chronic GVHD** | | **GRFS** | |
| --- | --- | --- | --- | --- | --- | --- | --- | --- | --- | --- | --- | --- | --- | --- |
|  | HR (95% CI) | p value | HR (95% CI) | p value | HR (95% CI) | p value | HR (95% CI) | p value | HR (95% CI) | p value | HR (95% CI) | p value | HR (95% CI) | p value |
| **UD 9/10 (reference)** | **1** |  | **1** |  | **1** |  | **1** |  | **1** |  | **1** |  | **1** |  |
| **SCB** | **1.52 (1.08-2.14)** | **0.017** | **2.54 (1.73-3.74)** | **<10-4** | **1.97 (1.54-2.52)** | **<10-4** | **2.02 (1.56-2.62)** | **<10-4** | **1.13 (0.81-1.56)** | **0.48** | **0.98 (0.67-1.43)** | **0.91** | **1.71 (1.36-2.14)** | **< 0.0001** |
| **DCB** | **1.24 (0.89-1.73)** | **0.2** | **1.74 (1.18-2.56)** | **0.005** | **1.45 (1.14-1.86)** | **0.003** | **1.44 (1.11-1.86)** | **0.007** | **1.48 (1.09-2.01)** | **0.011** | **0.9 (0.63-1.29)** | **0.58** | **1.31 (1.05-1.64)** | **0.019** |
| Age (per 10y) | 0.99 (0.9-1.08) | 0.76 | 1.3 (1.17-1.44) | **<10-4** | 1.12 (1.05-1.2) | 0.001 | 1.18 (1.1-1.27) | **<10-4** | 1.11 (1.02-1.21) | 0.013 | 1.06 (0.96-1.17) | 0.25 | 1.07 (1.01-1.14) | 0.033 |
| CR1 (reference) | 1 |  | 1 |  | 1 |  | 1 |  | 1 |  | 1 |  | 1 |  |
| CR2+ | 1.09 (0.79-1.5) | 0.61 | 1.33 (1-1.78) | 0.053 | 1.21 (0.98-1.5) | 0.074 | 1.28 (1.03-1.6) | 0.029 | 1.04 (0.81-1.34) | 0.76 | 1.08 (0.8-1.46) | 0.62 | 1.14 (0.94-1.39) | 0.18 |
| advanced | 2.82 (2.12-3.75) | **<10-4** | 1.94 (1.4-2.68) | **<10-4** | 2.29 (1.86-2.82) | < 0.0001 | 2.39 (1.92-2.97) | **<10-4** | 0.8 (0.58-1.1) | 0.18 | 0.95 (0.61-1.49) | 0.82 | 1.75 (1.43-2.14) | < 0.0001 |
| Good risk AML (reference) | 1 |  | 1 |  | 1 |  | 1 |  | 1 |  | 1 |  | 1 |  |
| Int. risk AML | 1.6 (0.77-3.32) | 0.21 | 0.81 (0.48-1.36) | 0.43 | 1.08 (0.71-1.64) | 0.71 | 1.07 (0.7-1.65) | 0.75 | 1.21 (0.74-1.98) | 0.44 | 0.75 (0.45-1.24) | 0.26 | 0.94 (0.66-1.34) | 0.72 |
| Adverse risk AML | 3.87 (1.83-8.18) | 4,00E-04 | 0.77 (0.43-1.4) | 0.39 | 1.77 (1.13-2.75) | 0.012 | 1.52 (0.96-2.42) | 0.077 | 1.35 (0.79-2.31) | 0.27 | 0.69 (0.37-1.26) | 0.22 | 1.38 (0.94-2.02) | 0.1 |
| missing | 1.93 (0.92-4.03) | 0.081 | 0.86 (0.51-1.47) | 0.59 | 1.24 (0.81-1.89) | 0.32 | 1.22 (0.79-1.89) | 0.38 | 1.3 (0.78-2.16) | 0.32 | 0.88 (0.52-1.5) | 0.64 | 1.19 (0.83-1.71) | 0.34 |
| Female vs male | 0.92 (0.72-1.17) | 0.51 | 0.85 (0.67-1.07) | 0.17 | 0.88 (0.74-1.04) | 0.12 | 0.88 (0.74-1.05) | 0.16 | 0.83 (0.68-1.02) | 0.078 | 0.9 (0.7-1.15) | 0.39 | 0.82 (0.7-0.96) | 0.012 |
| RIC vs MAC | 1.34 (1.01-1.77) | 0.041 | 0.66 (0.5-0.88) | 0.005 | 0.95 (0.78-1.15) | 0.57 | 0.88 (0.72-1.08) | 0.23 | 0.84 (0.65-1.08) | 0.17 | 0.88 (0.65-1.2) | 0.41 | 0.92 (0.76-1.1) | 0.34 |
| KPS≥90 | 0.96 (0.73-1.27) | 0.77 | 0.81 (0.61-1.06) | 0.13 | 0.89 (0.73-1.08) | 0.23 | 0.89 (0.73-1.08) | 0.24 | 0.74 (0.58-0.94) | 0.014 | 1.19 (0.86-1.65) | 0.29 | 0.86 (0.72-1.03) | 0.096 |
| center (frailty) |  | 0.33 |  | 0.072 |  | 0.28 |  | 0.27 |  | 0.003 |  | 0.002 |  | 0.18 |

Abbreviations: SCB- single cord blood, DCB- double cord blood, MMUD- mismatched unrelated donor; NRM-nonrelapse mortality; LFS-leukemia-free survival; OS-overall survival; GVHD- graft-vs-host disease; GRFS-GVHD, relapse free survival; HR-hazard ratio; CI-confidence interval

Table S6: Transplant outcomes in different subgroups

**Disease status at transplant:**

|  | **CR1** | | |
| --- | --- | --- | --- |
|  | MMUD (n=179) | CBT (n=522) | P value |
| Relapse | 20.3%[14.1-27.5] | 24.9%[21.1-28.9] | 0.34 |
| NRM | 15.1%[9.8-21.4] | 27.2%[23.3-31.3] | 0.003 |
| LFS | 64.6%[56-72] | 47.9%[43.2-52.3] | 0.002 |
| OS | 68.4%[59.9-75.5] | 53.1%[48.4-57.6] | 0.003 |
| GRFS | 52.1%[43.5-60] | 37.5%[33.1-41.9] | 0.003 |
| Acute GVHD II-IV | 32.7%[25.7-39.8] | 38.1%[33.9-42.3] | 0.12 |
| chronic GVHD | 31.4%[23.5-39.5] | 28.4%[24.4-32.6] | 0.57 |
|  |  |  |  |
|  | **CR2+** | | |
|  | MMUD (n=49) | CBT (n=230) | P value |
| Relapse | 21.7%[10.6-35.4] | 20.5%[15.3-26.2] | 0.88 |
| NRM | 15.2%[6.5-27.3] | 31.8%[25.7-38.1] | 0.04 |
| LFS | 63%[46.6-75.6] | 47.7%[40.8-54.2] | 0.08 |
| OS | 60.8%[42.5-74.9] | 51.1%[44.2-57.7] | 0.1 |
| GRFS | 41.7%[25.9-56.9] | 38.5%[32-45] | 0.29 |
| Acute GVHD II-IV | 23.4%[12.5-36.3] | 38.7%[32.1-45.2] | 0.041 |
| chronic GVHD | 42.2%[25.8-57.7] | 27.4%[21.5-33.6] | 0.09 |
|  |  |  |  |
|  | **active disease** | | |
|  | MMUD (n=52) | CBT (n=150) | P value |
| Relapse | 35.2%[20.8-49.9] | 47.4%[38.8-55.5] | 0.17 |
| NRM | 21.6%[10.9-34.8] | 35.2%[27.3-43.1] | 0.14 |
| LFS | 43.1%[27.7-57.6] | 17.4%[11.5-24.4] | 0.008 |
| OS | 44.9%[28.5-59.9] | 17.8%[11.8-24.9] | 0.01 |
| GRFS | 33.5%[19.7-47.8] | 13.6%[8.4-20.2] | 0.013 |
| Acute GVHD II-IV | 35.3%[22.4-48.4] | 24.5%[17.7-31.9] | 0.2 |
| chronic GVHD | 21.4%[8.9-37.6] | 15.2%[9.4-22.4] | 0.65 |

**Year of transplant:**

|  | **<2016** | | |
| --- | --- | --- | --- |
|  | **UD 9/10 (89)** | **CBT (729)** | **P value** |
| Relapse | 27.1%[17.9-37.2] | 27%[23.7-30.4] | 0.94 |
| NRM | 16.6%[9.5-25.4] | 30.8%[27.3-34.2] | 0.011 |
| LFS | 56.2%[44.7-66.3] | 42.2%[38.4-45.9] | 0.02 |
| OS | 61.4%[49.7-71.1] | 45.7%[41.8-49.4] | 0.009 |
| GRFS | 42.4%[31.5-52.8] | 32.5%[29-36.1] | 0.031 |
| Acute GVHD II-IV | 32.6%[22.9-42.6] | 35.4%[31.8-38.9] | 0.41 |
| chronic GVHD | 35.4%[24.8-46.2] | 26.7%[23.4-30.2] | 0.11 |
|  |  |  |  |
|  | **≥2016** | | |
|  | **UD 9/10 (89)** | **CBT (729)** | **P value** |
| Relapse | 20.8%[14.5-27.8] | 30.2%[22.5-38.2] | 0.16 |
| NRM | 16.2%[10.8-22.6] | 24.7%[18.1-31.9] | 0.08 |
| LFS | 63%[54.4-70.4] | 45.1%[36.5-53.4] | 0.014 |
| OS | 63.6%[54.5-71.4] | 52.3%[43.6-60.3] | 0.07 |
| GRFS | 49.4%[40.7-57.6] | 39.8%[31.6-47.9] | 0.21 |
| Acute GVHD II-IV | 31.2%[24.6-38] | 38.8%[31.3-46.2] | 0.09 |
| chronic GVHD | 29.9%[21.9-38.4] | 23.4%[16.6-30.9] | 0.31 |

**MRD status before HCT**

|  | **MRD negative** | | | **MRD positive** | | |
| --- | --- | --- | --- | --- | --- | --- |
|  | **MMUD (n=59)** | **CBT (n=165)** | **P value** | **MMUD (N=38)** | **CBT (N=71)** | **P value** |
| Relapse | 19.4%[9.8-31.3] | 17.4%[11.6-24.1] | 0.67 | 19%[7.5-34.6] | 31%[20.3-42.3] | 0.23 |
| NRM | 5.3%[1.4-13.5] | 27%[20.1-34.5] | 0.002 | 8.7%[2.1-21.3] | 24.8%[15.2-35.6] | 0.07 |
| LFS | 75.3%[61.1-84.9] | 55.6%[47-63.3] | 0.025 | 72.3%[53.1-84.7] | 44.2%[32-55.6] | 0.015 |
| OS | 75.3%[58.9-85.9] | 58.4%[49.7-66.1] | 0.019 | 74.3%[54.7-86.4] | 52.1%[39.3-63.4] | 0.049 |
| GRFS | 59.7%[43.7-72.5] | 44.9%[36.6-52.8] | 0.033 | 45.3%[27.4-61.7] | 35.5%[24.2-46.9] | 0.52 |
| Acute GVHD II-IV | 30.2%[18.7-42.6] | 37.2%[29.6-44.7] | 0.21 | 35.1%[20.2-50.5] | 33.8%[22.6-45.4] | 0.95 |
| chronic GVHD | 30.4%[17.1-44.7] | 29.3%[21.9-37.2] | 0.88 | 46%[26.8-63.2] | 21.9%[12.6-32.8] | 0.032 |

***In vivo* T cell depletion**

|  | **no in vivo TCD** | | |
| --- | --- | --- | --- |
|  | **MMUD (N=206)** | **CBT (N=543)** | **P value** |
| Relapse | 23.2%[17-30] | 28.3%[24.4-32.3] | 0.27 |
| NRM | 13.2%[8.6-18.7] | 24.1%[20.5-27.9] | 0.003 |
| LFS | 63.6%[55.6-70.5] | 47.6%[43.1-51.9] | 0.001 |
| OS | 65.3%[57-72.5] | 52.2%[47.7-56.5] | 0.003 |
| GRFS | 47.1%[39-54.8] | 37.5%[33.3-41.7] | 0.011 |
| Acute GVHD II-IV | 33.5%[27-40.1] | 43.7%[39.4-47.9] | 0.003 |
| chronic GVHD | 32%[24.3-39.9] | 26.3%[22.4-30.3] | 0.37 |
|  |  |  |  |
|  | **in vivo TCD** | | |
|  | **MMUD (N=74)** | **CBT (N=359)** | **P value** |
| Relapse | 23.2%[13.5-34.3] | 26.1%[21.4-31.1] | 0.6 |
| NRM | 24%[14.4-35] | 38.7%[33.3-44] | 0.038 |
| LFS | 52.8%[39.7-64.3] | 35.2%[29.9-40.6] | 0.02 |
| OS | 56.7%[43.4-67.9] | 38%[32.5-43.5] | 0.014 |
| GRFS | 46.2%[33.6-57.9] | 28.2%[23.3-33.3] | 0.009 |
| Acute GVHD II-IV | 25.9%[16.2-36.7] | 24.5%[20.1-29.2] | 0.86 |
| chronic GVHD | 30.7%[19.7-42.5] | 26.1%[21.2-31.1] | 0.36 |

Table S7: Cox with *in vivo* TCD and Year of HSCT

|  | **Acute GVHD II-IV** | | **chronic GVHD** | | **RELAPSE** | | **NRM** | | **LFS** | | **OS** | | **GRFS** | |
| --- | --- | --- | --- | --- | --- | --- | --- | --- | --- | --- | --- | --- | --- | --- |
|  | HR (95% CI) | p value | HR (95% CI) | p value | HR (95% CI) | p value | HR (95% CI) | p value | HR (95% CI) | p value | HR (95% CI) | p value | HR (95% CI) | p value |
| CBT vs MMUD | 1.55 (1.11-2.16) | **0.009** | 0.89 (0.61-1.3) | 0.55 | 1.31 (0.93-1.84) | 0.13 | 1.85 (1.26-2.74) | **0.002** | 1.55 (1.2-1.99) | **0.0008** | 1.55 (1.18-2.03) | **0.002** | 1.41 (1.12-1.78) | **0.004** |
| Patient age (per 10 years) | 1.13 (1.03-1.23) | 0.007 | 1.07 (0.96-1.18) | 0.21 | 0.99 (0.91-1.09) | 0.87 | 1.3 (1.17-1.44) | < 10^-4^ | 1.12 (1.05-1.2) | 0.0007 | 1.19 (1.11-1.28) | < 0.0001 | 1.08 (1.01-1.15) | 0.018 |
| CR1 (reference) | 1 |  | 1 |  | 1 |  | 1 |  | 1 |  | 1 |  | 1 |  |
| CR2 | 1 (0.77-1.29) | 0.97 | 1.07 (0.79-1.44) | 0.66 | 1.05 (0.76-1.45) | 0.77 | 1.33 (0.99-1.78) | 0.057 | 1.2 (0.97-1.49) | 0.092 | 1.27 (1.01-1.59) | 0.038 | 1.12 (0.92-1.36) | 0.25 |
| advanced | 0.85 (0.62-1.18) | 0.33 | 0.95 (0.6-1.5) | 0.83 | 2.94 (2.21-3.93) | < 10^-4^ | 1.86 (1.34-2.58) | 0.0002 | 2.29 (1.85-2.84) | < 10^-4^ | 2.43 (1.94-3.04) | < 0.0001 | 1.8 (1.47-2.21) | < 10^-4^ |
| Good risk AML (reference) | 1 |  | 1 |  | 1 |  | 1 |  | 1 |  | 1 |  | 1 |  |
| Int. risk AML | 1.18 (0.72-1.94) | 0.5 | 0.74 (0.45-1.24) | 0.26 | 1.58 (0.76-3.28) | 0.22 | 0.85 (0.51-1.43) | 0.54 | 1.11 (0.73-1.68) | 0.63 | 1.1 (0.71-1.7) | 0.66 | 0.94 (0.66-1.34) | 0.74 |
| Adverse risk AML | 1.32 (0.77-2.27) | 0.31 | 0.69 (0.37-1.26) | 0.22 | 3.76 (1.78-7.93) | 0 ;0005 | 0.8 (0.44-1.44) | 0.46 | 1.77 (1.14-2.76) | 0.011 | 1.52 (0.95-2.42) | 0.078 | 1.36 (0.92-2) | 0.12 |
| Unknown risk AML | 1.45 (0.87-2.43) | 0.16 | 0.87 (0.51-1.48) | 0.6 | 1.89 (0.9-3.96) | 0.091 | 0.83 (0.49-1.42) | 0.5 | 1.19 (0.78-1.83) | 0.41 | 1.16 (0.75-1.82) | 0.5 | 1.16 (0.81-1.68) | 0.41 |
| Female vs male | 0.83 (0.68-1.02) | 0.08 | 0.9 (0.7-1.15) | 0.4 | 0.94 (0.74-1.19) | 0.6 | 0.84 (0.66-1.07) | 0.16 | 0.89 (0.75-1.05) | 0.16 | 0.89 (0.75-1.07) | 0.21 | 0.83 (0.71-0.97) | 0.016 |
| RIC vs MAC | 0.72 (0.56-0.94) | 0.015 | 0.85 (0.62-1.15) | 0.29 | 1.22 (0.93-1.6) | 0.15 | 0.65 (0.49-0.87) | 0.003 | 0.89 (0.74-1.08) | 0.25 | 0.83 (0.68-1.02) | 0.08 | 0.83 (0.7-1) | 0.05 |
| KPS≥90 | 0.73 (0.57-0.93) | 0.011 | 1.18 (0.85-1.63) | 0.33 | 0.94 (0.71-1.24) | 0.65 | 0.79 (0.6-1.04) | 0.093 | 0.86 (0.71-1.05) | 0.13 | 0.85 (0.7-1.05) | 0.12 | 0.84 (0.7-1.01) | 0.068 |
| **Year of HSCT** | 1.02 (0.97-1.07) | 0.53 | 0.98 (0.93-1.04) | 0.57 | 0.98 (0.93-1.04) | 0.47 | 0.98 (0.92-1.03) | 0.4 | 0.98 (0.94-1.02) | 0.33 | 0.98 (0.94-1.02) | 0.35 | 0.98 (0.94-1.01) | 0.25 |
| ***in vivo* TCD** | 0.44 (0.33-0.58) | < 10^-4^ | 0.94 (0.68-1.3) | 0.71 | 0.88 (0.67-1.16) | 0.37 | 1.41 (1.06-1.88) | 0.018 | 1.18 (0.98-1.43) | 0.088 | 1.22 (0.99-1.5) | 0.062 | 0.95 (0.79-1.15) | 0.61 |
| centre (frailty) |  | 0.001 |  | 0.001 |  | 0.68 |  | 0.096 |  | 0.32 |  | 0.23 |  | 0.099 |

Table s8: Cox model including HCT-CI

|  | **CB vs MMUD** | |
| --- | --- | --- |
| **Outcomes** | HR (95% CI) | p value |
| RI | 1.15 (0.8-1.65) | 0.46 |
| NRM | 2.24 (1.46-3.43) | 0.0002 |
| LFS | 1.61 (1.23-2.1) | 0.0004 |
| OS | 1.69 (1.25-2.29) | 0.0006 |
| GRFS | 1.41 (1.09-1.84) | 0.01 |
| Acute GVHD II-IV | 1.44 (1.02-2.05) | 0.039 |
| Chronic GVHD | 1.18 (0.77-1.81) | 0.45 |

Table s9: outcomes by degree of HLA-match

|  | **9/10 MMUD (n=280)** | **CBT < 2 HLA mis-matches (n=224)** | **CBT ≥ 2 HLA mis-matches (n=331)** | **P value** |
| --- | --- | --- | --- | --- |
| Relapse | 23.2%[17.9-29] | 28.7%[22.6-35.1] | 28.2%[23.3-33.3] | 0.43 |
| NRM | 16.3%[11.9-21.3] | 25.3%[19.6-31.3] | 29.6%[24.6-34.7] | 0.002 |
| LFS | 60.5%[53.7-66.6] | 46%[39-52.7] | 42.2%[36.6-47.7] | 0.001 |
| OS | 62.8%[55.8-69] | 52.3%[45.1-58.9] | 45.9%[40.2-51.4] | 0.001 |
| GRFS | 46.8%[40-53.3] | 36.9%[30.3-43.4] | 32.7%[27.5-38] | 0.002 |
| Acute GVHD, grade II-IV | 31.6%[26.1-37.2] | 39%[32.4-45.5] | 37.5%[32.2-42.8] | 0.08 |
| Chronic GVHD | 31.5%[25.1-38] | 26.8%[20.7-33.2] | 27.2%[22.3-32.4] | 0.65 |

Number of mismatches: A & B at the antigenic level, DR at the allelic level. For double CBT, the highest number of HLA mismatches between the two CBU was considered. There were 347 CBT patients with missing HLA information.

Table s10: Different schedules and dose of PTCy

| Drug | Dose and schedule |
| --- | --- |
| PTCy | 50 mg/kg, Day +3, +4 |
| PTCy | 50 mg/kg, Day +3, +5 |
